# Epidermotropism of T-lymphocytes may provide insight into the pathogenesis of morphea

**DOI:** 10.1016/j.jdin.2026.01.011

**Published:** 2026-02-05

**Authors:** Lauren Khoury, Adrien Osakwe, Fadi Touma, Elvis Martinez-Jaramillo, Stephanie Ghazal, Ajay Rajaram, Mohammed Kaouache, Abdulmalik Alqahtani, Ozge Kizilay, May Chergui, Philippe Lefrançois, Yue Li, Ivan V. Litvinov, Elena Netchiporouk

**Affiliations:** a Faculty of Medicine, McGill University, Montreal, Quebec, Canada; b Quantitative Life Sciences Program, McGill University, Montreal, Quebec, Canada; c Division of Dermatology, Department of Medicine McGill University, Montreal, Quebec, Canada; d Department of Pathology, McGill University Health Center and McGill University, Montreal, Quebec, Canada; e Department of Mathematics and General Sciences, Prince Sultan University, Riyadh, Saudi Arabia; f Department of Pathology, Prince Sultan Military Medical City, Riyadh, Saudi Arabia; g School of Computer Science, McGill University, Montreal, Quebec, Canada

**Keywords:** clonality, digital pathology, epidermotropism, immunohistochemistry, localized scleroderma, morphea, T-lymphocytes

Morphea (localized scleroderma) is an immune-mediated fibrosing disorder that evolves from an early inflammatory phase to established sclerosis.^1^ During its inflammatory stage, morphea may exhibit histopathologic features that overlap with patch-stage cutaneous T-cell lymphoma (CTCL), particularly epidermotropism.^2^^,^^3^ When accompanied by T-cell receptor (TCR) clonality, epidermotropism may pose a diagnostic pitfall with important clinical consequences.^2^^,^^3^ However, the prevalence, immunophenotype, and diagnostic significance of epidermotropism and clonality in morphea remain incompletely defined.

We analyzed 64 skin biopsies from patients with clinicopathologically confirmed morphea evaluated at McGill University Health Center. Blinded histopathologic review was independently performed by 2 dermatopathologists to assess epidermotropism and disease stage, with inter-rater agreement evaluated and discordant cases resolved by consensus.^4^ Immunohistochemistry (CD3/4/8/45) with digital image analysis (QuPath) was used to quantify epidermal and dermal immune cell densities, and T cell receptor (TCR) clonality was assessed using standardized BIOMED-2 polymerase chain reaction assays.^5^ Statistical comparisons of continuous and categorical variables were performed using nonparametric and exact tests, with exploratory regression and receiver operating characteristic analyses where appropriate (Supplementary Methods, available via Mendeley at https://data.mendeley.com/datasets/thr22vjccm/1).

Epidermotropism was identified in 10/64 biopsies (15.6%), all from clinically active lesions, and was significantly enriched in the histologically inflammatory stage compared with fibrotic morphea (Table I and Table S1, available via Mendeley at https://data.mendeley.com/datasets/thr22vjccm/1; Figure S1, available via Mendeley at https://data.mendeley.com/datasets/thr22vjccm/1). Intraepidermal lymphocytes (CD45+, CD4+ > CD8+) displayed bland cytology, lacked Pautrier microabscesses, and lacked band-like lichenoid infiltrates, consistent with reactive epidermotropism rather than CTCL. Epidermal CD45+ cell density demonstrated the strongest association with epidermotropism among evaluated immune markers. An exploratory threshold of ≥143 CD45+ cells/mm^2^ identified epidermotropism with 90% sensitivity and 65% specificity (Table S1, Figure S2, available via Mendeley at https://data.mendeley.com/datasets/thr22vjccm/1). While not intended for clinical application, this quantitative benchmark illustrates the potential utility of objective metrics to complement subjective histologic assessment.

**Table I:** Demographics and clinical characteristics of patients with morphea, stratified by epidermotropism status

	Total	No epidermotropism	Epidermotropism∗	*P*
N	64	54	10	
Age (median [Q1, Q3])	59.0 [44.0, 65.2]	59.0 [42.5, 66.8]	56.5 [46.8, 64.5]	.781
Sex				.292
Male	22 (34.4%)	17 (31.5%)	5 (50.0%)	
Female	42 (65.6%)	37 (68.5%)	5 (50.0%)	
Subtype				.354
Circumscribed	25 (39.1%)	23 (42.6%)	2 (20.0%)	
Generalized	15 (23.4%)	13 (24.1%)	2 (20.0%)	
Linear	5 (7.81%)	4 (7.41%)	1 (10.0%)	
Not available (N/A)	19 (29.7%)	14 (25.9%)	5 (50.0%)	
Total CD45+ cell count (median [Q1, Q3])	244 [128, 593]	204 [126, 566]	696 [342, 1460]	**.007**†
Epidermis CD45+ cell count (median [Q1, Q3])	93 [38, 262]	74 [27, 217]	277 [177, 470]	**.002**†
Clinical activity status				.578
Active	58 (90.6%)	48 (88.9%)	10 (100%)	
Inactive	6 (9.38%)	6 (11.1%)	0 (0.00%)	
Histologic classification				**.048**†
Fibrotic	37 (57.8%)	34 (63.0%)	3 (30.0%)	
Inflammatory/fibrotic	4 (6.25%)	2 (3.70%)	2 (20.0%)	
Inflammatory	23 (35.9%)	18 (33.3%)	5 (50.0%)	

Continuous variables are reported as medians with interquartile ranges (Q1, Q3), while categorical variables are presented as frequencies with percentages. Statistical comparisons were performed to assess differences between groups. CD45+ cell count is reported as cells/mm^2^. Not available (N/A) indicates cases where consent to review detailed medical records was not obtained, and hence clinical details were unavailable.

∗ Initial inter-rater agreement was moderate (κ = 0.44, *P* < .001), resolving to perfect agreement after adjudication (κ = 1.00, *P* < .001).

† Statistically significant.

TCR clonality analysis (evaluable in 26 biopsies) demonstrated monoclonal rearrangements in 5 (19.2%) and partial clonality in 14 (53.8%) samples (Table S2, available via Mendeley at https://data.mendeley.com/datasets/thr22vjccm/1). Two clonal cases also exhibited epidermotropism, while 3 did not. All clonal cases occurred in clinically active, histologically inflammatory morphea in female patients. Notably, 1 patient with inflammatory *en coup de sabre* morphea was initially misclassified as CTCL due to the coexistence of epidermotropism and TCR clonality, resulting in delayed initiation of appropriate immunosuppressive therapy. A subsequent biopsy performed after disease evolution to a fibrotic stage showed resolution of epidermotropism and loss of clonality (Figure 1). Similar diagnostic confusion has been reported in prior cases of early morphea mimicking patch-stage mycosis fungoides.^3^

**Fig 1:**
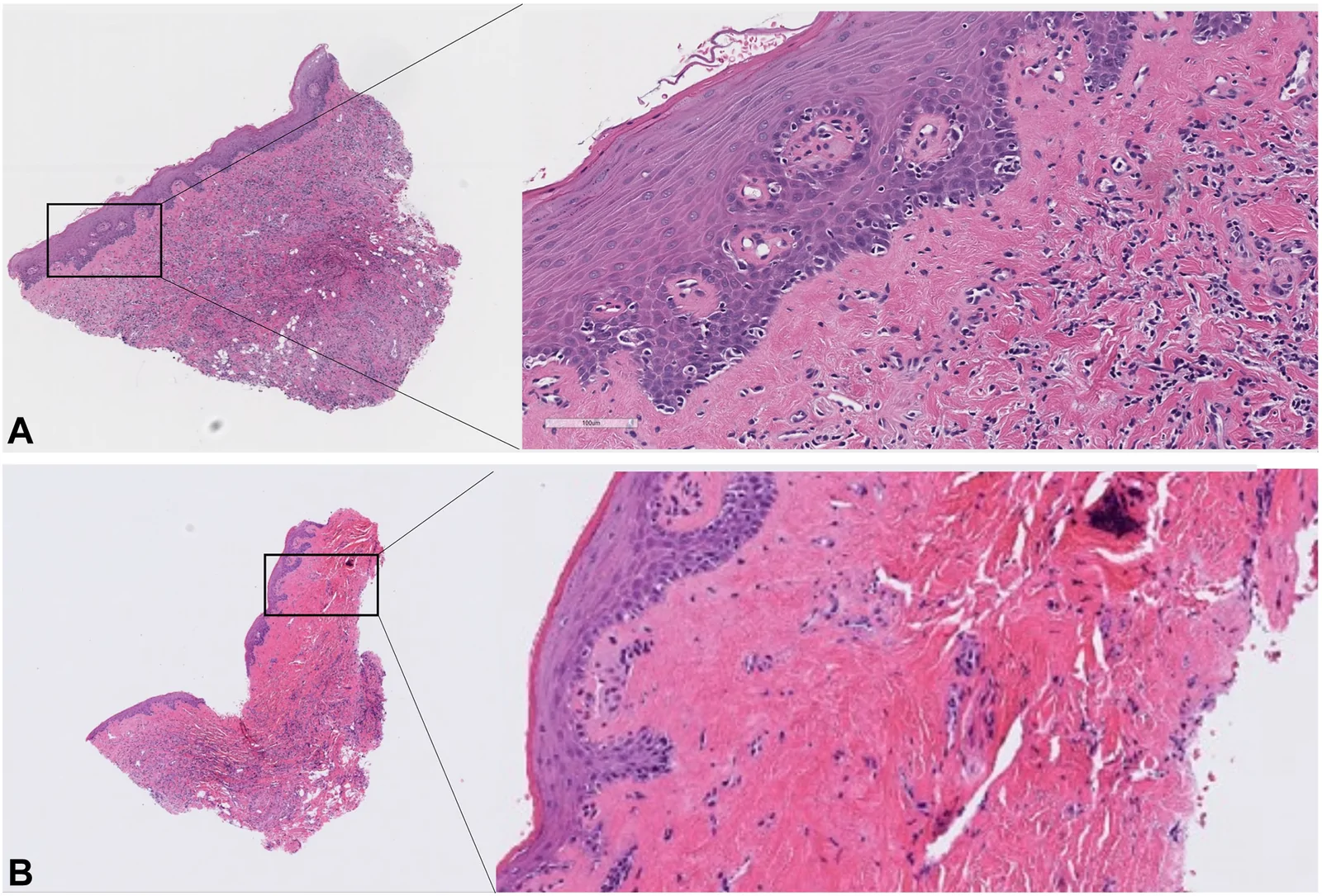
Temporal histopathologic variability demonstrating early and later-stage morphea. Medium-power visualization of the 2 biopsies of the same patient (upper cutaneous lip). **A,** Early-stage, inflammatory morphea with evident epidermotropism of CD3+, CD4+, CD8+, and CD45+ T cells. Clonality analysis revealed positive clones in all TCR-beta strategies and one TCR-gamma strategy. **B,** Later-stage (∼6 months later), mixed inflammatory/fibrotic morphea characterized by dermal sclerosis reduced inflammatory infiltrate, resolved epidermotropism and absence of clonality. The patient was referred to the skin fibrosis clinic and initiated methotrexate therapy. This case highlights the temporal variability of these findings and their restriction to the inflammatory phase.

These findings underscore that epidermotropism and T-cell clonality, although characteristic of CTCL, are not disease specific and may occur in inflammatory morphea. Comparable interpretive challenges arise in other inflammatory dermatoses, including lichen sclerosus, which can exhibit epidermotropic lymphocytes and clonal T-cell populations. Reported prevalence and features of epidermotropism and clonality across morphea and other inflammatory dermatoses are summarized in Table S3 (available via Mendeley at https://data.mendeley.com/datasets/thr22vjccm/1), while key clinicopathologic distinctions between morphea and CTCL are outlined in Table S4 (available via Mendeley at https://data.mendeley.com/datasets/thr22vjccm/1).

Our study is limited by modest sample size, incomplete clonality evaluability due to tissue quality, and the absence of CTCL or inflammatory comparator cohorts. Nevertheless, we show that epidermotropism and T-cell clonality in morphea are transient features of early inflammatory disease activity, rather than fixed markers of malignancy. Future studies could explore noninvasive approaches such as tape stripping to determine whether early epidermal immune signatures parallel biopsy findings and provide prognostic or longitudinal disease information.

## Conflicts of interest

None disclosed.
