# Supplementary material for: Epidermotropism of T-lymphocytes may provide insight into the pathogenesis of morphea
Source: JAAD Int. 2026 Feb 5;28:100–2. doi: 10.1016/j.jdin.2026.01.011 (PMC13584055; doi:10.1016/j.jdin.2026.01.011)
Supplement: Supplementary file 1 [file mmc1.docx]

## **Supplementary Methods**

### **Study design and setting**

We conducted a histopathologic and molecular study of formalin-fixed, paraffin-embedded (FFPE) skin biopsies collected between January 1, 2018 and May 16, 2022 at the McGill University Health Centre (MUHC). The protocol was approved by the MUHC Research Ethics Board, and archival tissue was reviewed under a consent waiver (#2021-7412). For patients with available consent, detailed clinical data were analyzed; otherwise, only limited demographic and histopathologic information was reviewed.

### **Participants and eligibility**

Biopsies were retrieved from patients with clinical suspicion of morphea. One patient contributed two biopsies from the same site at different times, representing early inflammatory and later fibrotic disease. Clinical data including age, sex, morphea subtype, and activity (active vs inactive) were abstracted from patient charts. None of the patients had concomitant lichen sclerosus.

### **Histopathologic assessment**

Hematoxylin and eosin (H&E)–stained sections were independently reviewed by two dermatopathologists (MC, AA), blinded to clinical information and immunostains. Morphea diagnosis was confirmed using established criteria, and biopsies were categorized as inflammatory, fibrotic, or mixed.[^1^](https://paperpile.com/c/5U5Kgv/jyqR) Epidermotropism was defined as the presence of intraepidermal lymphocytes in the absence of spongiosis or keratinocyte necrosis.[^2^](https://paperpile.com/c/5U5Kgv/f6vX) Inter-rater agreement was quantified with Cohen’s κ, with discordant cases resolved by consensus.[^3^](https://paperpile.com/c/5U5Kgv/9UO0)

### **Immunohistochemistry**

### Immunohistochemistry (IHC) was performed on 4-µm FFPE sections using the Discovery XT Autostainer (Ventana Medical Systems). Primary antibodies included CD45, CD3, CD4, and CD8 (Roche). All LS-confirmed samples underwent CD45 staining; 53 samples had sufficient tissue for CD3, CD4, and CD8 analysis.

### **Digital pathology and immune cell quantification**

### Whole-slide images were acquired from all IHC-stained sections. Epidermal and dermal compartments were manually annotated in QuPath (v0.4.0), and automated positive-cell detection algorithms were used to quantify immune cell densities (cells/mm²).[^4^](https://paperpile.com/c/5U5Kgv/h39k) Automated outputs were validated against manual counts on representative fields by a dermatopathologist.

### **T-cell receptor clonality analysis**

Genomic DNA was extracted from 52 biopsies with adequate tissue quality. T-cell receptor β (TCRB) and γ (TCRG) rearrangements were assessed by polymerase chain reaction (PCR) using standardized BIOMED-2 primers and protocols.[^5,6^](https://paperpile.com/c/5U5Kgv/Vubr+sqml) Amplicons were analyzed by capillary electrophoresis with GeneScan fluorescence detection.[^5^](https://paperpile.com/c/5U5Kgv/Vubr) Monoclonality was defined as a dominant peak of expected size, in line with CTCL diagnostic guidelines.[^7^](https://paperpile.com/c/5U5Kgv/brUs) Results were classified as monoclonal, partial clonality, negative, or uninterpretable (poor DNA yield).

### **Statistical Analysis**

Analyses were performed using R (v4.1.3) and GraphPad Prism (v10.4.1). Continuous variables were summarized as medians with interquartile ranges (IQR) and compared using Mann–Whitney U tests. Categorical variables were compared using Fisher’s exact test. Associations were examined with univariate logistic regression; odds ratios (OR) and 95% confidence intervals (CI) were reported. Correlations were assessed with Spearman’s ρ. Receiver operating characteristic (ROC) curves and area under the curve (AUC) were used to evaluate discriminative performance of immune markers.[^8^](https://paperpile.com/c/5U5Kgv/gjOb) An optimal epidermal CD45+ threshold was determined by Youden’s index.[^9,10^](https://paperpile.com/c/5U5Kgv/JnVm+8mqO) A two-sided p<0.05 was considered significant. Given the exploratory design, no correction for multiple comparisons was applied.

**References for Supplementary Methods**

**1.** Khoury L, Prosty C, Ghazal S, et al. Markers of type 2 inflammation and immunosenescence are upregulated in localized scleroderma. *Int J Mol Sci.* 2025;26(3):1258. doi:10.3390/ijms26031258

**2.** Fung MA. “Epidermotropism” vs. “exocytosis” of lymphocytes 101: definition of terms. *J Cutan Pathol.* 2010;37(5):525–529. doi:10.1111/j.1600-0560.2010.01515.x

**3.** McHugh ML. Interrater reliability: the kappa statistic. *Biochem Med (Zagreb).* 2012;22(3):276–282. doi:10.11613/bm.2012.031

**4.** Humphries MP, Maxwell P, Salto-Tellez M. QuPath: The global impact of an open source digital pathology system. *Comput Struct Biotechnol J.* 2021;19:852–859. doi:10.1016/j.csbj.2021.01.022

**5.** Beaubier NT, Hart AP, Bartolo C, Willman CL, Viswanatha DS. Comparison of capillary electrophoresis and polyacrylamide gel electrophoresis for the evaluation of T and B cell clonality by polymerase chain reaction. *Diagn Mol Pathol.* 2000;9(3):121–131. doi:10.1097/00019606-200009000-00001

**6.** van Dongen JJM, Langerak AW, Brüggemann M, et al. Design and standardization of PCR primers and protocols for detection of clonal immunoglobulin and T-cell receptor gene recombinations in suspect lymphoproliferations: report of the BIOMED-2 Concerted Action BMH4-CT98-3936. *Leukemia.* 2003;17(12):2257–2317. doi:10.1038/sj.leu.2403202

**7.** Olsen EA, Whittaker S, Kim YH, et al. Clinical end points and response criteria in mycosis fungoides and Sézary syndrome. *J Clin Oncol.* 2011;29(18):2598–2607. doi:10.1200/JCO.2010.32.0630

**8.** Carter JV, Pan J, Rai SN, Galandiuk S. ROC-ing along: Evaluation and interpretation of receiver operating characteristic curves. *Surgery.* 2016;159(6):1638–1645. doi:10.1016/j.surg.2015.12.029

**9.** Lai CY, Tian L, Schisterman EF. Exact confidence interval estimation for the Youden index and its corresponding optimal cut-point. *Comput Stat Data Anal.* 2012;56(5):1103–1114. doi:10.1016/j.csda.2010.11.023

**10.** Schisterman EF, Faraggi D, Reiser B, Hu J. Youden Index and the optimal threshold for markers with mass at zero. *Stat Med.* 2008;27(2):297–315. doi:10.1002/sim.2993

**Supplemental Table S1.** Predictors of Epidermotropism in Morphea.

| **Variables** | **Logistic Regression** | | **AUC, 95% CI^†^** |
| --- | --- | --- | --- |
|  | **OR, 95% CI** | ***P* value** |  |
| **CD3+** | 1.002 (1.001–1.005) | **0.010*** | 0.5756 (0.1841-0.9670) |
| **CD4+** | 1.010 (1.004–1.018) | **0.008*** | 0.6545 (0.3613-0.9477) |
| **CD8+** | 1.027 (1.008–1.059) | **0.032*** | 0.5930 (0.1887-0.9973) |
| **Total CD45+ Cell Count** | 2.82 (1.50 - 6.08) | **0.003*** | 0.7077 (0.4971-0.9183) |
| **Epidermis CD45+ Cell Count** | 2.11 (1.15-4.14) | **0.020*** | 0.7423 (0.5984-0.8862) |
| **Sex (Female)** | 0.46 (0.11-1.85) | 0.260 | — |
| **Age** | 1.05 (0.70-1.66) | 0.830 | — |

**Legend.** Odds ratios (OR) derive from univariate logistic-regression models; immune-marker densities and CD45+ counts are standardized (OR per 1 standard deviation increase). Age is scaled per 10-year increase. **P* <0.05 denotes statistical significance

†Leave-one-out cross-validation (LOOCV)-derived area under the curve (AUC) reported immune-marker predictors evaluated with receiver operating characteristic analysis (ROC) performance for immune markers.

**Supplemental Table S2.** T Cell Clonality in Morphea.

| **Clonality Status** | **n (%)** | **Definition** |
| --- | --- | --- |
| Monoclonal**^†^** | 5 (19.2) | Concordant TCRG/TCRB or dominant peak or TCRG (JP+) and negative TCRB |
| Partial clonality | 14 (53.8) | Positive in either T cell loci |
| Negative | 7 (26.9) | No clonal rearrangement detected in either TCRG or TCRB assays |
| Excluded (Low DNA) | 26 (50) | Poor DNA quality: results are uninterpretable |

**Legend.** Frequency (proportions) of epidermotropism classifications and expert-guided clonality classification of samples grouped by TCRG/TCRB panel results.

† Consistent with CTCL diagnostic criteria.

## **Supplementary Table S3.** Reported Prevalence and Characteristics of Epidermotropism and T-Cell Clonality in Morphea and Other Inflammatory Dermatoses

| **Disease** | **Epidermotropism prevalence** | **Epidermotropic cell features** | **T-cell clonality findings** | **Key references** |
| --- | --- | --- | --- | --- |
| Morphea | ~15% of biopsies | Bland cytology, CD3+, CD4>CD8+, absence of Pautrier microabscesses | Monoclonality in ~15% of evaluable cases; partial clonality frequent; usually phase-dependent (inflammatory > fibrotic) | this study |
| Systemic sclerosis | Not reported | Not reported | Oligoclonal/monoclonal expansions reported in blood and skin | Kreuter 2009  Fox 2021  Servaas 2021 |
| Lichen Sclerosus | Common | Bland cytology, CD3+, CD4+, CD8+, CD57+ | Monoclonal expansions reported in 49% samples | Lukowsky2000; Carlson 2000 |
| Lupus erythematosus | Present in some active/interface lesions | Bland, CD4/CD8 mixed, interface change | Oligoclonality (T and B cells) possible in chronic disease | Margo 2001  Liu 2019 |
| Lichen planus | Common, often dense band-like lichenoid | Bland, CD8+, basal keratinocyte damage | Monoclonality described | Li 2023 |
| Vitiligo | Common | Bland, CD8+, CD69+, CD103+, CXCR3+ | Monoclonal explansions in ~20% | Le Gal 2001; Boniface 2018 |
| CTCL (mycosis fungoides, Sézary) | By definition frequent, often prominent | Atypical, cerebriform lymphocytes; Pautrier microabscesses | Monoclonality in >90% (skin/blood), stable across time | Raghavan 2018; Iyer 2023 |

**References for Supplementary Table S3**

Systemic sclerosis
Kreuter A, Höxtermann S, Tigges C, Hahn SA, Altmeyer P, Gambichler T. Clonal T-cell populations are frequent in the skin and blood of patients with systemic sclerosis. *Br J Dermatol.* 2009;161(4):785–790. doi:10.1111/j.1365-2133.2009.09213.x

[Fox DA, Lundy SK, Whitfield ML, et al. Lymphocyte subset abnormalities in early diffuse cutaneous systemic sclerosis. *Arthritis Res Ther*. 2021;23(1):10. doi:](http://paperpile.com/b/5U5Kgv/egiS)[10.1186/s13075-020-02383-w](http://dx.doi.org/10.1186/s13075-020-02383-w)

[Servaas NH, Zaaraoui-Boutahar F, Wichers CGK, et al. Longitudinal analysis of T-cell receptor repertoires reveals persistence of antigen-driven CD4+ and CD8+ T-cell clusters in systemic sclerosis. *J Autoimmun*. 2021;117(102574):102574. doi:](http://paperpile.com/b/5U5Kgv/BBEf)[10.1016/j.jaut.2020.102574](http://dx.doi.org/10.1016/j.jaut.2020.102574)

Lichen sclerosus
Lukowsky A, Muche JM, Sterry W, Audring H. Detection of expanded T cell clones in skin biopsy samples of patients with lichen sclerosus et atrophicus by T cell receptor-gamma polymerase chain reaction assays. *J Invest Dermatol.* 2000;115(2):254–259. doi:10.1046/j.1523-1747.2000.00040.x

Carlson JA, Grabowski R, Chichester P, et al. Comparative immunophenotypic study of lichen sclerosus: epidermotropic CD57+ lymphocytes are numerous—implications for pathogenesis. *Am J Dermatopathol.* 2000;22(1):7–16. doi:10.1097/00000372-200002000-00002

Lupus erythematosus
Magro CM, Crowson AN, Kovatich AJ, et al. Lupus profundus, indeterminate lymphocytic lobular panniculitis and subcutaneous T-cell lymphoma: a spectrum of subcuticular T-cell lymphoid dyscrasia. *J Cutan Pathol.* 2001;28(5):235–247. doi:10.1034/j.1600-0560.2001.028005235.x

[Liu X, Zhang W, Zhao M, et al. T cell receptor β repertoires as novel diagnostic markers for systemic lupus erythematosus and rheumatoid arthritis. *Ann Rheum Dis*. 2019;78(8):1070-1078. doi:](http://paperpile.com/b/5U5Kgv/h1BY)[10.1136/annrheumdis-2019-215442](http://dx.doi.org/10.1136/annrheumdis-2019-215442)

Lichen planus
Li Q, Wang F, Shi Y, et al. Single-cell immune profiling reveals immune responses in oral lichen planus. *Front Immunol.* 2023;14:1182732. doi:10.3389/fimmu.2023.1182732

Vitiligo
Le Gal FA, Avril MF, Bosq J, et al. Direct evidence to support the role of antigen-specific CD8+ T cells in melanoma-associated vitiligo. *J Invest Dermatol.* 2001;117(6):1464–1470. doi:10.1046/j.0022-202x.2001.01605.x

Boniface K, Jacquemin C, Darrigade AS, et al. Vitiligo skin is imprinted with resident memory CD8 T cells expressing CXCR3. *J Invest Dermatol.* 2018;138(2):355–364. doi:10.1016/j.jid.2017.08.038

Cutaneous T-cell lymphoma (mycosis fungoides, Sézary syndrome)
Raghavan SS, Kim J. Histopathologic approach to epidermotropic lymphocytic infiltrates. *Semin Cutan Med Surg.* 2018;37(1):56–60. doi:10.12788/j.sder.2018.003

Iyer A, Hennessey D, O’Keefe S, et al. Independent evolution of cutaneous lymphoma subclones in different microenvironments of the skin. *Sci Rep.* 2020;10(1):15483. doi:10.1038/s41598-020-72459-9

**Supplementary Table S4.** Distinguishing Features of Morphea Versus Cutaneous T-Cell Lymphoma.

| **Feature** | **Morphea (localized scleroderma)** | **Cutaneous T-cell lymphoma (CTCL, mycosis fungoides)** |
| --- | --- | --- |
| **Typical clinical presentation** | Indurated plaques or linear bands with ivory sclerosis and lilac border; distribution may be circumscribed, generalized, or linear | Patch, plaque, or tumor-stage lesions; erythematous to violaceous patches often with poikiloderma, progressive over years |
| **Age/sex** | All ages; peaks in children and middle-aged adults; female predominance | Mostly adults >50 years; slight male predominance |
| **Course** | Inflammatory → fibrotic evolution; potential extracutaneous involvement (fascia, muscle, joint contractures) | Indolent but progressive; potential nodal/visceral spread in advanced disease |
| **Histopathology (inflammatory stage)** | Perivascular and periadnexal lymphocytic infiltrates; dermal edema; evolving dermal sclerosis | Superficial lichenoid or band-like infiltrate of atypical lymphocytes with epidermotropism |
| **Histopathology (fibrotic stage)** | Thickened, sclerotic collagen; loss of adnexal structures; sparse inflammation |  |
| **Epidermotropism** | Can occur, usually sparse, morphologically normal lymphocytes; no atypia | Hallmark finding; atypical cerebriform lymphocytes with haloed nuclei; frequent, sometimes forming Pautrier microabscesses |
| **T-cell clonality** | Detected in subset; usually reactive/oligoclonal; may resolve with disease progression | Detected in majority; stable dominant clone supports diagnosis |
| **Pertinent negatives** | No cytologic atypia; absence of lichenoid band-like infiltrate; dermal sclerosis extends beyond papillary dermis | Persistent epidermotropism with atypical lymphocytes; band-like infiltrates common; no dermal sclerosis |
| **Evolution / follow-up** | May improve or remit with immunosuppressive therapy; epidermotropism and clonality can disappear over time | Chronic, progressive; lesions accumulate and advance from patch to tumor stage; clonal persistence over time |

**Supplementary Figure S1.** Immunohistochemical Staining of Lymphocyte Markers in a Single Patient Biopsy.

**Legend.** Immunohistochemical analysis of a patient biopsy stained for lymphocyte markers: (A) CD3 (T cells), (B) CD4 (helper T cells), (C) CD8 (cytotoxic T cells) and (D) CD45+ cells. (E-H) Comparison of cell density estimates for CD3 (E), CD4 (F), CD8 (G) and (H) CD45 positive cells for samples with and without epidermotropism. Statistical significance was determined using the Mann-Whitney U test, with P-values represented as follows: p < 0.05 (*), p < 0.010 (**), p < 0.001 (***), p < 0.001 (****). Sample sizes for each group are indicated above the corresponding box plots.

**Supplementary Figure S2.** ROC Analysis and Youden’s Index for CD45+ cells.

**Legend:** (A) The receiver operating characteristic (ROC) curve for CD45-based Epidermotropism classification yields an area under curve (AUC) of 0.742. (B) The distribution of positive and negative examples as determined by consensus dermatopathologists, as well as the number of CD45+ cells in the epidermis (cells/mm^2^) as counted by QuPath. Youden's Index is calculated by finding the probability threshold that maximizes J=Sensitivity+Specificity−1 and provides the optimal cutoff value (143 cells/mm^2^). Sensitivity: 90%, Specificity: 65%, Youden’s Index: 0.55. (C) Diagram depicting how CD45+ cell detection via immunohistochemistry staining and cell detection algorithms such as QuPath could be used to facilitate the detection of Epidermotropism in LS patients.
